# How does staff and patient feedback on hospital quality relate to mortality outcomes? A provider-level national study

**DOI:** 10.1177/09514848231179182

**Published:** 2023-06-27

**Authors:** Antonio Michael Borrelli, Rebecca J Birch, Katie Spencer

**Affiliations:** 1 Sheffield Teaching Hospitals NHS Foundation Trust, Northern General Hospital, South Yorkshire, Sheffield S5 7AT, UK; 2 Leeds Institute for Data Analytics, 4468 University of Leeds, Leeds, UK

**Keywords:** Staff views, patient satisfaction, mortality, quality improvement

## Abstract

This study aimed to use national data to examine the relationship between staff and inpatient survey results (National Health Service (NHS) Friends and Family Test (FFT)) and assess how these align with more traditional measurements of hospital quality as captured by the summary hospital mortality indicator (SHMI). Provider level FFT responses were obtained for 128 English non-specialist acute providers for staff and inpatients between April 2016 and March 2019. Multilevel linear regression models assessed the relationship between staff and patient FFT recommendations, and separately how SHMI related to each of staff and patient FFT recommendations. A total of 1,536 observations were recorded across all providers and financial quarters. Patients were more likely to recommend their provider (95.5%) than staff (76.8%). In multivariable regression, a statistically significant association was observed between staff and patient FFT recommendations. A statistically significant negative relationship was also observed between staff FFT recommendations and SHMI. The association between SHMI and staff FFT recommendations suggests that staff feedback tools may provide a useful analogue for providers in potential need of intervention and improvement in care. For patients meanwhile, qualitative approaches and hospital organisations working in partnership with patients may provide better opportunities for patients to drive improvement.

## Introduction

Feedback on healthcare delivery, whether from patients or staff, is of considerable interest to healthcare providers. Principally this interest relates to the use of such feedback as a measure of healthcare quality. Staff feedback has been shown to be effective in screening hospital doctors providing poor levels of care in England (via the ‘Colleague Feedback Evaluation Tool’ (CFET)),^1^ whilst patient feedback in the form of the ‘Doctor’s Interpersonal Skills Questionnaire’ (DISQ) was found to be effective in the same study.^1^ Similarly, online patient feedback on primary care providers has been found to be associated with measures of healthcare quality in England^2^ and inspection outcomes by England’s healthcare regulator, the Care Quality Commission (CQC).^3^

Alongside understanding how feedback relates to care quality, an appetite also exists to utilise this feedback to facilitate quality improvement in hospitals.^4^ Managers have shown a desire to promote change in response to patient feedback across healthcare jurisdictions,^5,6^ although Lee et al.^6^ noted that different forms of feedback were not used as effectively as they could be to monitor and improve care quality. Nonetheless, patient feedback can help improve the performance of doctors, especially with respect to behavioural changes.^7^

Of importance to consider though, is the format in which feedback is received. While Boylan et al.^8^ found awareness and usage of online feedback to be increasing, those providing feedback using this format were rarely representative of the general population, generally being younger and more educated. Within the English National Health Service (NHS), the Friends and Family Test (FFT) was introduced in 2013 for hospitals across the NHS. It gives all patients treated by, and staff working in, every provider the opportunity to give anonymous feedback on the quality of care. Crucially, responses can be provided in several formats including paper, online, phone and text message. That this allows it to be more easily completed by a variety of patients, and is offered to all patients and staff across the English NHS, means it has potential to provide a more representative assessment than online only surveys are able to offer.

The FFT has the potential to provide near real time feedback to help drive quality improvement. An important first step in considering its potential to do so however, is to ensure it can accurately identify those providers most in need of intervention. Identifying how closely FFT results align with more traditional measurements of hospital quality such as the summary hospital mortality indicator (SHMI) provides one way in which this may be done. While SHMI may not necessarily be a reliable direct measure of healthcare quality, it can help identify variations in healthcare requiring intervention.^9^ Having a source of patient or staff feedback which is associated with risk-adjusted mortality measures like the SHMI may allow for earlier identification of providers in potential need of intervention and improvement in care.

Patient FFT responses have previously been studied in relation to hospital quality measures. Lawton et al.^10^ found patient responses to the FFT were not associated with actual safety outcomes, although this study included only 33 wards in three Northern English hospitals. In contrast, Greaves et al.^11^ identified a small, but statistically significant correlation between patient FFT responses and the SHMI for the first year the FFT was conducted. Although a national study, as with Lawton et al.,^10^ only patient FFT responses were considered. This study aimed to build on these previous works by considering the more extensive data now available, including assessing staff feedback alongside patient feedback. It examined, at a national level, the relationship between anonymous staff and inpatient FFT survey results, and how these align with a traditional measure of risk-adjusted mortality in the SHMI. In doing so, it sought to identify the potential for both staff and patient feedback tools such as the FFT to serve as a more ‘real time’ means of identifying healthcare providers potentially in need of intervention, and in doing so, help to drive improvement.

## Methods

The NHS is a single payer healthcare system, with NHS hospital providers delivering both acute and routine care to the English population free at the point of use. A minority of care is though, delivered by the independent sector. For these analyses, only NHS providers were considered, with data presented at the hospital provider level. All English non-specialist acute NHS providers were considered. These include both secondary and tertiary centres, but exclude organisations providing only specific services, for example children’s hospitals, cancer centres and cardiac hospitals. Staff responses relate to all individuals working in a particular provider, with patient responses coming from those admitted as inpatients to the hospital provider.

Publicly available data for staff and inpatient FFT responses were obtained at NHS provider level from NHS Digital.^12,13^ The FFT is voluntary for both staff and patients, but the opportunity to provide a response is offered to all. Responses are based on a Likert-type scale to the question: ‘*How likely are you to recommend < this organisation > to friends and family if they needed care or treatment?*’ with options of ‘extremely likely’, ‘likely’, ‘neither likely or unlikely’, ‘unlikely’, ‘extremely unlikely’ and ‘don’t know’. These analyses considered the proportion of staff or inpatient responses which were either ‘extremely likely or ‘likely’ to recommend the care provided.

Patient FFT responses are published monthly, while staff responses are published quarterly. Data were obtained to allow analyses for the 2016-17, 2017-18 and 2018-19 financial years (April to March). To support the comparison between patient and staff responses by financial quarter (Q) (Q1 – April – June, Q2 – July – September, Q3 – October – December, Q4 – January – March), monthly patient FFT recommendations were amalgamated to provide a quarterly result. The staff FFT is conducted as a standalone exercise in Q1, Q2 and Q4, but reported as part of the annual NHS Staff Survey in Q3. Data were extracted from the annual staff survey to complete the full year staff FFT responses. As with the FFT published results, these data are publicly available and were obtained from the National NHS Staff Survey Co-ordination Centre.^14^

The SHMI provides an important analogue for clinical outcomes across English non-specialist acute NHS providers. It is a ratio of actual patient mortality following hospitalisation against the expected mortality based on the known baseline characteristics of patients treated. It therefore provides a measure which includes inpatients across all hospital departments. Unlike FFT results, SHMI data is not published quarterly, but annually on a monthly rolling basis. English non-specialist acute NHS provider level SHMI data were obtained from NHS Digital.^15^

Three analyses were carried out, and aimed to address three questions:

- How do staff and patient responses to the FFT relate to each other?
- Are staff FFT recommendations associated with SHMI?
- Are patient FFT recommendations associated with SHMI?

The anonymous nature of FFT responses meant patient-level confounders were not able to be considered for analysis. The published aggregated results meanwhile, did not include potential provider-level confounders, such as organisation size to be included for analysis. Nonetheless, for each analysis univariable and multivariable linear regression models were assessed. Initially, a pooled regression was carried out. Subsequently, multilevel models were produced incorporating the clustering of surveys within providers, with year and quarter incorporated at the result level. Based on the results of the Breusch-Pagan Lagrange multiplier (BPL) (*p* ≤ 0.001 for all analyses presented in this study) and recognition of the clustered nature of the survey data, the multilevel regression model results are presented here. Pooled regression model outcomes are presented in supplementary material (Supplemental Digital Content 1). Analyses were conducted in STATA IC 15.

As data were publicly available and patient identification not possible, ethical approval was not required.

## Results

Quarterly data for 128 English non-specialist acute NHS providers operating at the end of the 2018-19 financial year were available across the three financial years, generating 1,536 observations. Two providers were excluded, having only been formed during the analysis period. Additionally, reporting of staff FFT recommendations were incomplete for 25 providers, affecting a total of 38 quarters (2.5% of results). For patient FFT recommendations, reporting was incomplete for one provider, affecting a single quarter (0.07% of results). These providers have been included in analyses, with affected quarters entered as missing values.

On average, patient FFT responses were more positive than staff, with 95.5% of patients recommending compared with 76.8% of staff. There was also less variability in responses from patients (range = 83.0%–99.8%, SD = 0.025) compared with staff (range = 36.4%–100.0%, SD = 0.110) (Table 1).

**Table 1.** Summary statistics for staff FFT recommendations, patient FFT recommendations, staff response rates and patient response rates.

Time period	Staff proportion recommended in FFT Mean/standard deviation/range	Patients proportion recommended in FFT Mean/standard deviation/range	Staff proportion responded to FFT Mean/standard deviation/range	Patients proportion responded to FFT Mean/standard deviation/range
All years (Apr 2016 – Mar 2019)	0.768 (0.110)	0.955 (0.025)	0.188 (0.164)	0.251 (0.093)
	(0.364–1.000)	(0.830–0.998)	(0.0001–0.770)	(0.024–0.678)
Apr 2016 – Mar 2017 (2016-17 FY)	0.768 (0.106)	0.954 (0.023)	0.183 (0.162)	0.250 (0.089)
	(0.436–1.000)	(0.854–0.991)	(0.002–0.770)	(0.056–0.541)
Apr 2017 – Mar 2018 (2017-18 FY)	0.768 (0.111)	0.956 (0.025)	0.190 (0.163)	0.252 (0.093)
	(0.364–1.000)	(0.830–0.998)	(0.0005–0.730)	(0.039–0.678)
Apr 2018 – Mar 2019 (2018-19 FY)	0.768 (0.114)	0.955 (0.026)	0.192 (0.169)	0.252 (0.096)
	(0.389–0.973)	(0.830–0.997)	(0.0001–0.720)	(0.024–0.524)
Q1 (Apr – Jun) all years combined	0.800 (0.096)	0.957 (0.023)	0.113 (0.085)	0.259 (0.094)
	(0.495–0.976)	(0.866–0.998)	(0.0002–0.451)	(0.034–0.591)
Q2 (Jul – Sep) all years combined	0.793 (0.106)	0.955 (0.025)	0.107 (0.085)	0.256 (0.095)
	(0.389–1.000)	(0.830–0.996)	(0.0001–0.449)	(0.056–0.678)
Q3 (Oct – Dec) all years combined	0.700 (0.096)	0.954 (0.025)	0.438 (0.081)	0.245 (0.090)
	(0.411–0.909)	(0.850–0.996)	(0.250–0.770)	(0.024–0.501)
Q4 (Jan – Mar) all years combined	0.782 (0.109)	0.955 (0.026)	0.107 (0.088)	0.246 (0.092)
	(0.364–0.979)	(0.830–0.996)	(0.0006–0.469)	(0.026–0.541)

FFT recommendations showed limited inter-annual variability. For staff, average recommendation remained at 76.8% for each of the three financial years. For patients, recommendations ranged from an average of 95.4% in 2016-17 to 95.6% in 2017-18. While patient recommendations remained relatively stable between quarters, staff recommendations fell to 70.0% in Q3 (October – December) compared with 80.0% in Q1 (Table 1).

Marked variations in FFT response rates were observed between providers for both staff and patients. On average, patients (25.1%) were more likely to answer the FFT than staff (18.8%), although staff responses increased to 43.8% for the annual staff survey in Q3 (Table 1). On univariable multilevel linear regression, a significant relationship between staff FFT recommendations and response rates was observed; for every 1% increase in response rate a 0.238% fall in recommendations was observed (95% confidence interval (CI) = −0.637–−0.476, *p* = 0.001). In contrast, a very small, but significant association between patient recommendations and patient FFT response rates was observed. As patient response rates increased so too did recommendations (coefficient (COEF) = 0.000, CI = −0.013–0.014, *p* = 0.040).

### Staff FFT recommendations and patient FFT recommendations

A significant relationship was observed on univariable linear regression; for each 1% increase in patient FFT recommendations, there was a 0.756% (CI = 0.435 – 1.077, *p* = 0.001) increase in staff FFT recommendations (Table 2). After multivariable adjustment for financial quarter and year this significant relationship persisted; each percentage increase in patient FFT recommendations was associated with a 0.495% (CI = 0.216 – 0.774, *p* = 0.001) increase in staff FFT recommendations (Table 2).

**Table 2.** Multilevel univariate and multivariate linear regression analysis assessing the relationship between staff FFT recommendations and patient FFT recommendations. Overall R2 = 0.180 for the multivariable analysis.

Variables	Univariable			Multivariable		
	Coefficient	*p*-value	95% confidence interval	Coefficient	*p*-value	95% confidence interval
Patient FFT recommendations	0.756	0.001	0.435–1.077	0.495	0.001	0.216–0.774
Quarter 1	—	—	—	—	—	—
Quarter 2	−0.007	0.131	−0.163–0.002	−0.006	0.183	−0.016–0.003
Quarter 3	−0.105	0.001	−0.114–−0.096	−0.104	0.001	−0.113–−0.095
Quarter 4	−0.017	0.001	−0.026–−0.008	−0.016	0.001	−0.026–−0.007
2016-17 financial year	—	—	—	—	—	—
2017-18 financial year	0.000	0.960	−0.009–0.010	−0.001	0.879	−0.009–0.007
2018-19 financial year	−0.001	0.864	−0.011–0.009	−0.001	0.854	−0.009–0.007

### SHMI and Staff FFT recommendations

A statistically significant negative association was observed on univariable multilevel linear regression, with increasing staff FFT recommendations associated with a lower SHMI, and so mortality compared with expected mortality (COEF = −0.022, CI = −0.044 – −0.000, *p* = 0.047) (Table 3). This relationship persisted on multivariable analysis, with adjustment for quarter, year and response rate (COEF = −0.031, CI = −0.579 – −0.004, *p* = 0.023) (Table 3). Quarter, year and response rate showed no significant relationship with SHMI.

**Table 3.** Multilevel univariate and multivariate linear regression analysis results for SHMI and staff FFT recommendations. Overall R2 = 0.041 for the multivariable analysis.

Variables	Univariable			Multivariable		
	Coefficient	*p*-value	95% confidence interval	Coefficient	*p*-value	95% confidence interval
Staff FFT recommendations	−0.022	0.047	−0.044–−0.000	−0.031	0.023	−0.579–−0.004
Quarter 1	—	—	—	—	—	—
Quarter 2	0.000	1.000	−0.005–0.005	0.000	0.932	−0.004–0.005
Quarter 3	0.000	1.000	−0.005–0.005	0.002	0.744	−0.008–0.011
Quarter 4	0.000	1.000	−0.005–0.005	0.000	0.882	−0.004–0.005
2016-17 financial year	—	—	—	—	—	—
2017-18 financial year	−0.001	0.497	−0.005–0.003	−0.001	0.550	−0.005–0.003
2018-19 financial year	−0.002	0.236	−0.006–0.002	−0.003	0.206	−0.007–0.001
Staff response rates	−0.002	0.722	−0.013–0.009	−0.012	0.328	−0.037–0.012

### SHMI and Patient FFT recommendations

In contrast to the staff FFT recommendations, no significant associations were found between SHMI and patient FFT recommendations in a univariable analysis. A multivariable model with quarter, year and patient response rate was also performed. As with the univariable regression, results were not statistically significant, and have been included in Table 4.

**Table 4.** Multilevel univariable and multivariable linear regression analysis results for SHMI and patient FFT recommendations. Overall R2 = 0.003 for the multivariable analysis.

Variables	Univariable			Multivariable		
	Coefficient	*p*-value	95% confidence interval	Coefficient	*p*-value	95% confidence interval
Patient FFT recommendations	−0.0837	0.261	−0.230–0.0622	−0.0793	0.291	−0.226–0.0679
Quarter 1	—	—	—	—	—	—
Quarter 2	0.000	1.00	−0.00455–0.00455	−0.0000763	0.974	−0.00463–0.00448 0.00485
Quarter 3	0.000	1.00	−0.00455–0.00455	0.000152	0.948	−0.00445–0.00475
Quarter 4	0.000	1.00	−0.00455–0.00455	0.0001125	0.962	−0.00448–0.00470
2016-17 financial year	—	—	—	—	—	—
2017-18 financial year	−0.00136	0.497	−0.00530–0.00257	−0.00127	0.528	−0.00522–0.00268
2018-19 financial year	−0.00238	0.236	−0.00632–0.00155	−0.00240	0.233	−0.00634–0.001545
Patient response rates	0.0251	0.207	−0.0138–0.0640	0.0259	0.198	−0.0135–0.065336

## Discussion

The results of this study have demonstrated in a health service wide analysis that while staff and patient FFT recommendations are associated, only staff recommendations have a significant relationship with standardised hospital mortality. These associations suggest staff may provide a useful analogue for providers in need of intervention and improvement in care, and organisations could benefit from encouraging greater staff completion of a relatively simple survey. In contrast, patient surveys like the FFT were not demonstrated to have a significant relationship with SHMI, and thus are less suited to being used to identify providers potentially requiring improvement. Nonetheless, the positive association identified between staff and patient recommendations suggested both groups shared similar feelings towards their respective provider in a given time period. That associations between SHMI and staff FFT recommendations remained significant in univariable and multivariable multilevel analyses suggests a robust relationship, even if the effect size is modest.

Compared with the staff survey, patient responses were consistently more positive. This contradicted Abuosi^16^ and Alhassan et al.’s^17^ findings in Ghana. In part, this may be down to structural and financial differences in the healthcare systems. Other potential reasons may be social or demographic.^18^ Patient age has particularly been positively associated with satisfaction.^19,20^ Quintana et al.^19^ further found lower education levels, being married and male to be associated with greater patient satisfaction. Young et al.^20^ meanwhile, observed lower levels of patient satisfaction in larger hospitals and among more seriously ill patients. As an anonymous survey, such data on patient background are not captured within the survey, and so could not be used to provide additional variables. More detailed information on providers were also not presented in the raw data limiting more detailed analysis. However, the influence of both on patient feedback may form an area for further research.

The positive association between SHMI and staff FFT recommendations suggests the potential for greater use of staff feedback as a more real time means of identifying providers where care may be poor and improvement intervention potentially required. The reasons for FFT recommendations are not explored in the survey, and so were not investigated. However, Australian hospital workers have identified staff attitudes and behaviours contributing to whether they would recommend care.^21^ Additionally, with nurse staffing having been identified as a factor in both staff satisfaction and mortality,^22,23^ better staff understanding of shortages may provide a possible reason for the significant association between SHMI and staff recommendations. Given SHMI provides a relatively poor direct measure of care quality,^9^ associations between staff FFT recommendations and SHMI identified in this study means staff feedback surveys like the FFT should not be seen as a direct analogue of care quality. To understand the reasoning for poor staff feedback more fully, and identify required interventions, subsequent research, likely more qualitative in nature, would be required.

Providers should nonetheless consider providing greater encouragement to complete staff surveys like the FFT, and place greater emphasis on their results. Were the staff FFT to be used more by providers or other agencies though, the relationships between recommendations and response rates identified in this study may be important to consider. Response rates in Q3 were considerably higher as part of the staff survey, with average recommendations lower. This suggests possible response bias in FFT only quarters, with those less satisfied opting to simply not complete the survey. If there were to be greater encouragement to increase response rates, there may be a need to revalidate the associations identified in this study. Alongside this, there may also be a need to consider which staff members typically respond to the FFT rather than just through the annual staff survey. With the FFT being anonymous, it was not possible to evaluate this, and it is something the literature has thus far not explored. However, it may be another factor influencing the nature of the observed relationship.

For patients meanwhile, the results suggest the FFT may not be a particularly suitable feedback tool for identifying providers potentially in need of improvement. That is not to say though, that patient feedback is not important to improve care. Indeed, patient and public involvement is required for all such research, and has ensured patient-reported outcomes remain patient centred.^24^ Given the overwhelming proportion of patients recommending their provider in the FFT, it is possible the FFT question as a measure of overall satisfaction is too general, and so difficult to answer.

That the FFT is too vague has been a source of critique. Contradictory responses have sometimes been noted among patients which may create diverging responses, with complaints being made alongside a positive FFT response.^25^ This may have contributed to the lack of association found in this study. In primary care meanwhile, concerns have been raised around the FFT’s ability to identify specific episodes of good or poor-quality care, making it difficult to identify specific areas of improvement.^26^ These findings further suggest that patient feedback tools based on more specific aspects of care, with a greater emphasis on co-development, may be a more appropriate form of patient feedback than the FFT.

Among the strengths of the FFT, is that patient feedback can be easily aggregated and interpreted. In desiring easily analysable data however, providers are potentially failing to collect sufficient information to make meaningful changes. Marsh et al.^27^ identified 37 feedback tools across four categories; ‘hospital-initiated quantitative surveys’, ‘patient-initiated qualitative feedback’ (including formal complaints and social media comments, ‘hospital-initiated qualitative feedback’ and ‘other’, which includes the FFT. While they found few tools provided easily usable data for analysis, ‘hospital-initiated quantitative surveys’ such as the adult inpatient survey may be used to produce aggregated data. Such surveys are important in better understanding the patient experience. The FFT question, as asked, provides high level information regarding overall satisfaction with the care received. It fails, however, to more fully understand the patient experience, that is, the patient perspective on specific aspects of the care they have received. With Otani et al.^28^ identifying factors such as respect as important to patient satisfaction, it may be that there is greater value in focusing on experience than satisfaction when seeking patient feedback.

In primary care, better understanding the patient experience has been found to be more important for driving change than simple measures of satisfaction.^29^ To that end, the value of qualitative approaches to patient feedback are increasingly being utilised by providers, particularly in online forums. For one provider, online feedback not only improved accessibility and ease of use, but also allowed for feedback from stakeholders beyond the patient, the sharing of feedback in near real time, and greater dialogue between staff and patients to help drive change.^30^ The idea of online qualitative feedback creating a dialogue between patients and staff was also noted by Powell et al.,^31^ but is something lacking in current FFT methodology.

While Skillen^32^ has demonstrated how quantitative and qualitative data can be effectively combined in the FFT, the value of understanding patient experience has to some extent been seen with changes to the FFT methodology. Alongside changing the FFT question to focus on overall experience, a locally selected follow-up question will also be employed.^33^ In particular, this may provide the opportunity for organisations to focus on patient centredness aspects of the hospital experience. At the heart of the patient experience aspect of the Organisation for Economic Cooperation and Development’s (OECD) Health Care Quality Indicators (HCQI) are questions around patient centredness, something which was reaffirmed in the most recent revision of the framework.^34^ Including such questions may therefore ultimately help the patient FFT better gauge perceptions of specific aspects of patient-centred care, and drive improvement in this area, rather than be used as a high-level tool to ‘flag’ providers in need of improvement.

This study has many strengths. Chief among them is the approach, aided by the structure of the English NHS as a single payer healthcare system providing universal coverage, which allowed all responses across included providers to be considered. Despite potential response bias, the FFT is an important feedback tool for assessing the quality of care being provided, and all staff and patients could anonymously express their views through it. While the views expressed may not be representative of all patients or staff, these are nonetheless the data on which providers identify areas requiring improvement. The study’s approach was therefore effective in identifying limitations, particularly of the patient FFT, in predicting mortality outcomes. Additionally, the use of multilevel analyses recognised the clustering of outcomes within providers.

The study has limitations however, one of which being missing FFT responses. While minimal for patient recommendations, staff reporting was incomplete for 2.5% of all reports. Assuming these results are missing not at random (MNAR) the multilevel model structure should be robust to this missingness. It is, however, plausible that missing data may not be MNAR (i.e. providers with worse performance might systematically not contribute data). The impact of this could not be quantified, although it was likely minimal given the small proportion of missing observations. Finally, organisational changes may have impacted the analyses. The available data were unable to address the impact of organisational changes such as smaller providers being merged into larger ones without a change in larger provider name.

This study benefits from a health service wide analysis. If generalisable to other healthcare jurisdictions, similar staff surveys conducted in other healthcare systems may offer valuable information about healthcare providers. While the similar demographics, staff training and patient services in many countries with advanced healthcare systems would point to good generalisability, the results of this study could potentially be built upon with similar analyses in other countries. If replicated, staff surveys may provide a valuable, simple and near real time means of identifying providers where care may be poor and improvement intervention potentially required. For patient feedback meanwhile, more qualitative approaches, with hospitals and patients working in partnership, could provide better opportunities for patients to drive improvements in care. Further research would, however, be required to verify whether such approaches provide a more appropriate means by which patients can make their voices heard.

## Supplemental Material

Supplemental Material - How does staff and patient feedback on hospital quality relate to mortality outcomes? A provider-level national study Supplemental Material for How does staff and patient feedback on hospital quality relate to mortality outcomes? A provider-level national study by Antonio Michael Borrelli, Rebecca J Birch and Katie Spencer in Health Services Management Research.
