# Supplementary material for: How does staff and patient feedback on hospital quality relate to mortality outcomes? A provider-level national study
Source: Health Serv Manage Res. 2023 Jun 27;37(2):115–22. doi: 10.1177/09514848231179182 (PMC11041065; doi:10.1177/09514848231179182)
Supplement: Supplemental Material - How does staff and patient feedback on hospital quality relate to mortality outcomes? A provider-level national study [file sj-pdf-1-hsm-10.1177_09514848231179182.pdf]

Supplemental Information – pooled regression results

Supplementary Table 1: pooled univariate and multivariate linear regression analysis results for staff FFT recommendations and patient FFT recommendations. Adjusted R^2 was 0.184 for the multivariable analysis

Variables	Univariable			Multivariable		
	Coefficient	p-value	95% confidence interval	Coefficient	p-value	95% confidence interval
Patient FFT recommendations	0.810	0.001	0.730 – 1.179	0.891	0.001	0.684 – 1.099
Quarter 1	-	-	-	-	-	-
Quarter 2	-0.007	0.349	-0.022 – 0.008	-0.006	0.429	-0.020 – 0.009
Quarter 3	-0.105	0.001	-0.119 – -0.090	-0.102	0.001	-0.116 – -0.088
Quarter 4	-0.017	0.019	-0.032 – -0.003	-0.016	0.029	-0.020 – -0.002
2016-17 financial year	-	-	-	-	-	-
2017-18 financial year	0.000	0.974	-0.013 – 0.014	-0.001	0.815	-0.014 – 0.011
2018-19 financial year	-0.000	0.965	-0.014 - 0.013	-0.001	0.900	-0.013 – 0.012

Supplementary Table 2: pooled univariate and multivariate linear regression analysis results for SHMI and staff FFT recommendations. Adjusted R^2 was 0.041 for the multivariable analysis

Variables	Univariable			Multivariable		
	Coefficient	p-value	95% confidence interval	Coefficient	p-value	95% confidence interval
Staff FFT recommendations	-0.156	0.001	-0.201 – -0.111	-0.173	0.001	-0.222 – -0.004
Quarter 1	-	-	-	-	-	-
Quarter 2	0.000	1.000	-0.014 – 0.014	-0.001	0.851	-0.015 – 0.013
Quarter 3	0.000	1.000	-0.014 – 0.014	0.020	0.122	-0.005 – 0.044
Quarter 4	0.000	1.000	-0.014 – 0.014	-0.002	0.821	-0.016 – 0.012
2016-17 financial year	-	-	-	-	-	-
2017-18 financial year	-0.001	0.826	-0.014 – 0.011	-0.002	0.809	-0.014 – 0.011
2018-19 financial year	-0.002	0.702	-0.015 – 0.010	-0.002	0.808	-0.014 – 0.011
Staff response rates	-0.043	0.006	-0.074 – -0.012	-0.119	0.001	-0.178 – -0.059

Supplementary Table 3: pooled univariate and multivariate linear regression analysis results for SHMI and patient FFT recommendations. Overall R^2 was 0.006 for the multivariable analysis

**Variables**	**Univariable**			**Multivariable**		
	Coefficient	P-value	95% confidence interval	Coefficient	P-value	95% confidence interval
Patient FFT recommendations	0.141	0.171	-0.061 – 0.343	0.143	0.166	-0.059 – 0.344
Quarter 1	-	-	-	-	-	-
Quarter 2	0.000	1.000	-0.014 – 0.014	0.001	0.939	-0.014 – 0.015
Quarter 3	0.000	1.000	-0.014 – 0.014	0.002	0.797	-0.00445 – 0.00475
Quarter 4	0.000	1.000	-0.014 – 0.014	0.002	0.815	-0.012 – 0.016
2016-17 financial year	-	-	-	-	-	-
2017-18 financial year	-0.001	0.826	-0.014 – 0.011	-0.002	0.753	-0.014 – 0.010
2018-19 financial year	-0.002	0.702	-0.015 – 0.010	-0.003	0.651	-0.015 – 0.009
Patient response rates	0.102	0.001	0.048 – 0.155	0.102	0.001	0.049 – 0.156
